# Packaging protein drugs as bacterial inclusion bodies for therapeutic applications

**DOI:** 10.1186/1475-2859-11-76

**Published:** 2012-06-11

**Authors:** Antonio Villaverde, Elena García-Fruitós, Ursula Rinas, Joaquin Seras-Franzoso, Ana Kosoy, José Luis Corchero, Esther Vazquez

**Affiliations:** 1 Institut de Biotecnologia i de Biomedicina, Universitat Autònoma de Barcelona, Bellaterra 08193, Barcelona, Spain; 2 Departament de Genètica i de Microbiologia, Universitat Autònoma de Barcelona, Bellaterra 08193, Barcelona, Spain; 3 CIBER de Bioingeniería, Biomateriales y Nanomedicina (CIBER-BBN), Barcelona, Spain; 4 Institute of Technical Chemistry-Life Science, Leibniz University of Hannover, Hannover 30167, Germany; 5 Helmholtz Centre for Infection Research, Inhoffenstraße 7, Braunschweig D-38124, Germany; 6 Janus Development SL, Parc Científic Barcelona, Torre R, Baldiri Reixach 4, Barcelona 08028, Spain

## Abstract

A growing number of insights on the biology of bacterial inclusion bodies (IBs) have revealed intriguing utilities of these protein particles. Since they combine mechanical stability and protein functionality, IBs have been already exploited in biocatalysis and explored for bottom-up topographical modification in tissue engineering. Being fully biocompatible and with tuneable bio-physical properties, IBs are currently emerging as agents for protein delivery into mammalian cells in protein-replacement cell therapies. So far, IBs formed by chaperones (heat shock protein 70, Hsp70), enzymes (catalase and dihydrofolate reductase), grow factors (leukemia inhibitory factor, LIF) and structural proteins (the cytoskeleton keratin 14) have been shown to rescue exposed cells from a spectrum of stresses and restore cell functions in absence of cytotoxicity. The natural penetrability of IBs into mammalian cells (reaching both cytoplasm and nucleus) empowers them as an unexpected platform for the controlled delivery of essentially any therapeutic polypeptide. Production of protein drugs by biopharma has been traditionally challenged by IB formation. However, a time might have arrived in which recombinant bacteria are to be engineered for the controlled packaging of therapeutic proteins as nanoparticulate materials (nanopills), for their extra- or intra-cellular release in medicine and cosmetics.

## 

Since the full acknowledgment of bacterial inclusion bodies (IBs) as formed by functional polypeptides [1,2], enzyme-based IBs have been exploited as naturally immobilized catalysts with high operational stability [3,4]. Pull-down peptides, incorporated to target proteins as end-terminal fusions, favor the deposition of properly folded polypeptides in *Escherichia coli* as functional IBs [5-7]. This is especially relevant as these tags can drive protein deposition even under production conditions that favor protein folding (eg. suboptimal growth temperature), then enriching IBs with biologically active polypeptides [1,8-10].

Being mechanically stable, purified IBs have been recently observed as promising nanoparticulate materials [3,11-16], whose biological and nanoscale properties can be modulated by the appropriate selection of the *E. coli* host strain and of production/handing conditions [3]. In particular, IBs have been explored as agents for topographical modification in tissue engineering [11,17-19]. Being bio-adhesive, they favor mammalian cell attachment to IB-decorated surfaces but also offer convenient mechanical effectors within the mammalian cell sensing range that stimulate ERK-mediated cell proliferation [17]. No signs of toxicity or cell apoptosis have been ever observed in these studies. Previously reported toxicity on mammalian cells upon exposure to high amounts of IBs [20] could be linked to obsolete purification protocols leaving IBs contaminated with living bacterial cells or toxic debris. Interestingly, in bottom-up IB decoration, the mammalian cell membrane is in intimate contact with IBs [11] and cell sensing agents (filopodia/lamelipodia) are stimulated in presence of substrate IBs [17].

Taken together, the relatively cost-efficient production/downstream of IBs in *E. coli*, their biological activity [10], the tunability of their biological and nano-mechanical properties [3], their biocompatibility in cell interfaces [18,19,21], the release of functional IB proteins in aqueous conditions [8,22] and the apparent avidity of IBs for mammalian cell membranes [11,17] drives to the intriguing question about if these protein particles could deliver embedded therapeutic proteins into mammalian cells. If so, these bacterially produced nanoparticles could act as nanopills, that is, nanosized clusters of functional and bioavailable protein drugs. Recombinant *E. coli* cells would then turn into convenient factories for the tailored packaging of protein drugs as nanopills, since essentially any protein (with or without therapeutic potential) can be produced as bacterial IBs [23]. The same limitations defining the suitability of soluble proteins produced in *E. coli* as biopharmaceuticals (eg. biological activity depending on post-translational modifications, missing in bacteria, or proteolytic instability) would be relevant to bacterial nanopills.

The response to this exciting question was disclosed to be positive in 2010 [24]. Upon plain addition to the culture media, Hsp70 IBs prevented cis-platinum-induced apoptosis. In a recent follow-up of this pioneering report [25], rescue of cell viability has been observed when exposing serum-starving cells to leukemia inhibitory factor (LIF) IBs in absence of any sign of toxicity. Also, dihydrofolate reductase (DHFR) IBs were able to complement DHFR cell deficiency, and catalase (CAT) IBs rescued mammalian cells from oxidative stress [25]. To account for the observed protein replacement effect, especially in the case of the intracellular acting DHFR and Hsp70, IBs have to cross the cell membrane. Indeed, and depending on the IB-forming protein, between 35% and 70% of exposed cells fully (and naturally) internalize bacterial nanopills resuspended in the culture media 4 h after exposure (Figure 1A). Even more, images of IBs reaching the cell nucleus were common under confocal microscopy observations (Figure 1B), although the fraction of IB protein accumulating in the nuclear compartment remains to be quantitatively determined. In a more recent study published in Microbial Cell Factories [26], IBs formed by keratin 14 (K14) restore the formation of cytoskeleton in K14-deficent cells and, expectedly, the cell mechanical properties. In this case, electroporation facilitated intracellular delivery of K14 IBs.

**Figure 1:**
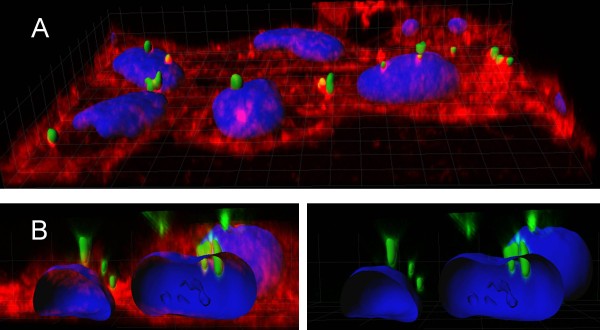
**A. 40-Section confocal xyz stack of HeLa cells exposed to GFP IBs, showing IB cell penetrability.** Cell membrane is labeled in red and the nuclear material is seen in blue. IBs are observed under their natural green fluorescence as discrete particulate entities. **B.** GFP IBs embedded or crossing the nuclear membrane are shown in two stack versions, in which the cell membrane is either shown (left) or hidden (right) for clarity. Modified from reference [25] (Copyright Wiley-VCH Verlag GmbH & Co, KGaA. Reproduced with permission).

The precise mechanisms by which IBs get naturally embedded and cross both cellular and nuclear membranes should be investigated, but we might anticipate that hydrophobic, solvent-exposed protein patches in IBs might have a role in there, as it occurs with cell penetrating peptides of common use for intracellular drug delivery [27]. Also, how functional proteins are released from IBs once in the cytoplasmic and nuclear compartments deserves additional analysis, to set a basis for further improvement of IB properties through protein or process engineering. A recent model proposing a cotton-like structure for bacterial IBs [13] figures out IBs as mainly composed by releasable soluble protein, entrapped into the gaps of a more stable scaffold.

In this regard, and being natural products, bacterial IBs are not homogeneous in their compositional analysis. IBs are in general almost exclusively formed by the target protein with little contamination of other proteins [12,28,29]. There are also strong indications that upon co-expression of different aggregation-prone proteins these species do not co-aggregate, but deposit into distinguishable IBs [30,31]. However, truncated versions of the target protein [32,33], other plasmid-encoded proteins [34,35], but also defined host cell proteins [34,36] including folding assistant proteins [36-39] may get entrapped within or associated to bacterial IBs. Mostly, the majority of host cell and plasmid derived contaminants (e.g. plasmid DNA, lipids, membrane components) in IB preparations reflect unspecific adsorption and co-precipitation of cell debris during IB purification [33]. Most of these contaminants can be removed by thorough purification procedures [40-42], and new protocols for IB purification have been recently communicated that permit to obtain these particles relatively free from contaminating cell debris, and specially from living bacteria escaping from cell lysis [41,42].

As amyloidal versions of hormones are natural reservoirs for slow release of proteins in mammalian tissues and organs [43,44], respective hormones produced in form of IBs using recombinant bacterial expression systems may also represent a versatile form for sustained protein delivery. However, the diversity of protein categories so far successfully administered as bacterial nanopills (enzymes, chaperones, structural proteins and grow factors) prompt us to propose the Nanopill concept as a generic emerging platform for drug delivery and protein-based cell therapy. In this regard, recombinant bacteria would be used as factories not only for protein production but also for protein packaging as nanostructured entities for further delivery (Figure 2). Furthermore, as we have shown that high IBs doses are well tolerated by mice models upon oral administration [25], further exploration of bacterial nanopills for innovative therapies *in vivo* will benefit from a solid starting point. How the potential uses of IBs in emerging medicines will be bounded by regulatory constrictions cannot be currently anticipated (again, being these particles heterogeneous natural products). However, pharmaceutical companies are facing critical challenges in reducing R&D expenses and they pursue the incorporation of new and innovative drugs, since their marketed products are reaching patent life expiration. The Nanopill system would open complete new research and market fields, complementary to the conventional multibillion dollar therapeutic protein-drug business currently in force.

**Figure 2:**
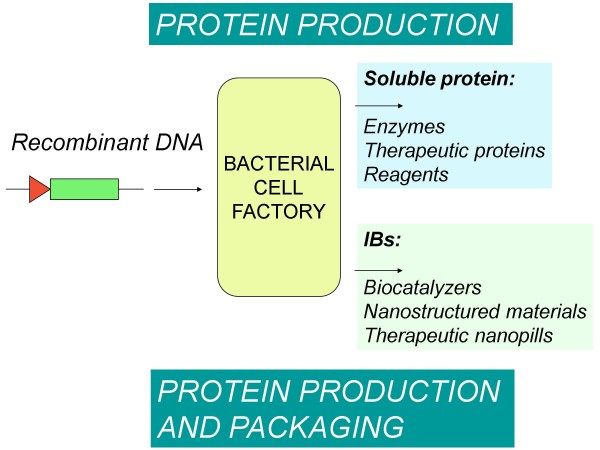
**Recombinant bacteria are conventionally exploited to produce soluble proteins for both Biotechnological and Pharmaceutical industries.** Alternatively, recombinant bacteria can be observed as protein production-packaging factories whose products are nanostructured proteinaceous entities formed by functional species (IBs). Despite their limitations, IBs show a spectrum of properties that make them appealing as immobilized catalysts and as biocompatible materials in tissue engineering. The revealing of the therapeutic potential of bacterial IBs as nanopills for protein replacement cell therapy expands the opportunities for the development and tailoring of IBs as desired bioproducts with commercial value.

## Conclusions

Bacterial IBs show a great and unexpected potential as cost-effective protein delivery agents. Available genetic and process tools permit the tailoring of relevant IB properties and prompt an immediate investigation of the new opportunities offered by IBs as nanopills, for advanced therapies in translational and innovative medicines.

## Authors’ contributions

All authors have read and approved the final version.

## Competing interests

EV, JLC, EGF and AV are co-inventors of a patent (WO2010131117A1) covering the use of IBs as protein delivery agents, currently licensed to Janus Development SL.
